# Antioxidant and Cytoprotective Effects of Kukoamines A and B: Comparison and Positional Isomeric Effect

**DOI:** 10.3390/molecules23040973

**Published:** 2018-04-21

**Authors:** Xican Li, Jian Lin, Ban Chen, Hong Xie, Dongfeng Chen

**Affiliations:** 1 School of Chinese Herbal Medicine, Guangzhou University of Chinese Medicine, Guangzhou 510006, China; helloban@foxmail.com (B.C.); xiehongxh1@163.com (H.X.); 2 Innovative Research & Development Laboratory of TCM, Guangzhou University of Chinese Medicine, Guangzhou 510006, China; 3 School of Basic Medical Sciences, Guangzhou University of Chinese Medicine, Guangzhou 510006, China; linjianchn@outlook.com; 4 Research Center of Basic Integrative Medicine, Guangzhou University of Chinese Medicine, Guangzhou 510006, China

**Keywords:** positional isomeric effect, antioxidant mechanisms, cytoprotective effect, kukoamine A, kukoamine B, phenolic polyamine

## Abstract

In this study, two natural phenolic polyamines, kukoamine A and B, were comparatively investigated for their antioxidant and cytoprotective effects in Fenton-damaged bone marrow-derived mesenchymal stem cells (bmMSCs). When compared with kukoamine B, kukoamine A consistently demonstrated higher IC_50_ values in PTIO•-scavenging (pH 7.4), Cu^2+^-reducing, DPPH•-scavenging, •O_2_^−^-scavenging, and •OH-scavenging assays. However, in the PTIO•-scavenging assay, the IC_50_ values of each kukoamine varied with pH value. In the Fe^2+^-chelating assay, kukoamine B presented greater UV-Vis absorption and darker color than kukoamine A. In the HPLC–ESI–MS/MS analysis, kukoamine A with DPPH• produced radical-adduct-formation (RAF) peaks (*m*/*z* 922 and 713). The 3-(4,5-Dimethylthiazol-2-yl)-2,5-diphenyl (MTT) assay suggested that both kukoamines concentration-dependently increased the viabilities of Fenton-damaged bmMSCs at 56.5–188.4 μM. However, kukoamine A showed lower viability percentages than kukoamine B. In conclusion, the two isomers kukoamine A and B can protect bmMSCs from Fenton-induced damage, possibly through direct or indirect antioxidant pathways, including electron-transfer, proton-transfer, hydrogen atom transfer, RAF, and Fe^2+^-chelating. Since kukoamine B possesses higher potentials than kukoamine A in these pathways, kukoamine B is thus superior to kukoamine A in terms of cytoprotection. These differences can ultimately be attributed to positional isomeric effects.

## 1. Introduction

**Figure 1:**
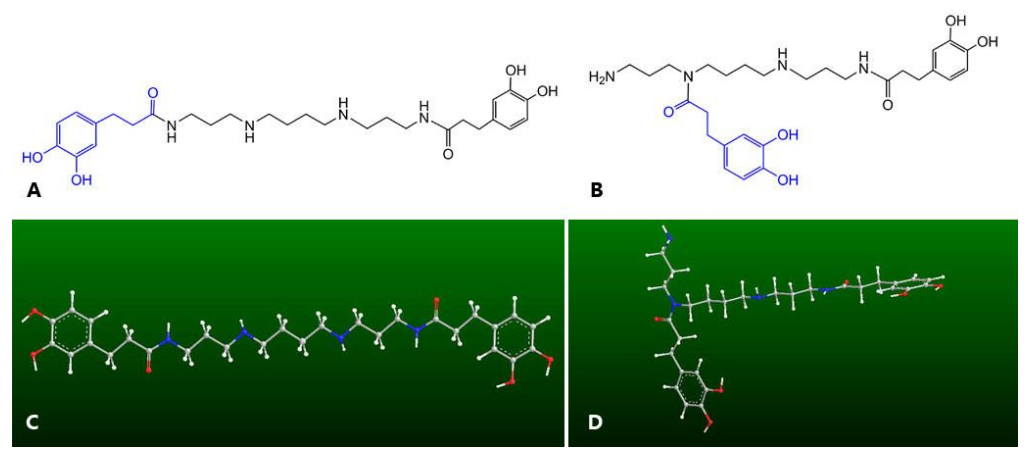
Structures (above) and preferential conformation-based ball-stick models (below) of kukoamine A (**A**,**C**) and kukoamine B (**B**,**D**). The ball-stick models were created using Chem3D Pro 14.0.

The majority of natural antioxidants are phenolic compounds, mainly including flavonoids, phenolic acids, tannins, coumarins, and anthraquinone [1,2], while none of these contain a nitrogen atom (N-atom) in their molecular scaffolds. In fact, natural antioxidants bearing a nitrogen atom are relatively rare in natural products. As phenolic alkaloids naturally present in the dried root bark of Lycium chinense, kukoamines A (KukA) and B (KukB) (Figure 1) however possess nitrogen atoms in their molecular structures, and they are actually spermine derivatives with dihydrocaffeoyl groups [3,4]. It is well known that dihydrocaffeic acid and their analogs are potent natural antioxidants with multiple mechanisms involving free radical scavenging and metal ion chelation [5]. Spermine is a natural antioxidant, which plays an important role in many cellular processes including protection of cells against oxidative damage by free radicals, and regulation of transcription and translation [5,6]. In recent years, increasing attention has been directed toward finding natural antioxidants for diseases associated with oxidative stress. Hence, kukoamine with two pharmacophores in the backbone structure is a promising candidate.

Accumulated evidence from studies in vivo and in vitro indicates the neuroprotection of kukoamines A and B against oxidative stress [7,8,9,10,11]. These neuroprotective effects have been reported to be closely related to antioxidant action, because oxidative damage induced by reactive oxygen species (ROS) is one of the main sources of neurotoxicity. To our knowledge, there is little literature on the antioxidant mechanisms of kukoamines A and B so far.

As shown in Figure 1, kukoamines A and B are actually isomers of each other, and the only difference between these two compounds is the position of dihydrocaffeoylation. Kukoamine A has a linear-chain structure in which two dihydrocaffeoyl moieties are individually connected to two terminal N-atoms; as for kukoamine B, one of the two dihydrocaffeoyl moieties is linked to one middle N-atom, which forms a branched chain structure. Such positional isomerism differs considerably from that of other phenols, e.g., ferulic acid and isoferulic acid. Ferulic acid is 3-hydroxy-4-methoxycinnamic acid; whereas isoferulic acid is 4-hydroxy-3-methoxycinnamic acid. Such small differences cannot change the molecular shape of these molecules. Therefore, there is no great difference in antioxidant levels between ferulic acid and isoferulic acid [12].

In addition to the molecular shape, another point to be noted is that kukoamine A and kukoamine B vary greatly in the relative distance of functional groups. As seen in Figure 1, the distance between the two dihydrocaffeoyl moieties is farther in kukoamine A with a linear-chain structure than that in kukoamine B. More importantly, the linear distance from the dihydrocaffeoyl moiety to the N-atom is nearer in kukoamine B (branched chain structure) than that in kukoamine A. Thereby, the dihydrocaffeoyl moiety and the N-atom in kukoamine B may interact with each other. This is well documented as a positional isomeric effect [13]. In fact, similar isomeric effects have been reported to influence molecular crystallization, morphology, chirality, and phototoxicity [14,15]. Nevertheless, there is no evidence regarding isomeric effect (especially positional isomeric effect) in antioxidants.

In this study, two phenolic alkaloids, kukoamines A and B, were comparatively investigated using a cellular model and several typical antioxidant assays. The cellular model is based on bone marrow-derived mesenchymal stem cells (bmMSCs) under oxidative stress. bmMSCs are considered a highly promising source for cell-based tissue engineering and stem cell transplantation, but the poor viability of transplanted cells caused by oxidative stress has been a bottleneck in the clinical application of MSC transplantation [16]. Obviously, this study will be of great significance. Based on the structural characteristics and bioactivity of kukoamine, the study will provide a novel candidate for MSC transplantation therapy and many diseases involved in oxidative stress. It will also contribute to better understand the antioxidant mechanisms of kukoamine, as well as other spermine derivatives or phenolic alkaloids. Moreover, this work will give evidence of (positional) isomeric effect in antioxidants.

## 2. Results and Discussion

**Figure 2:**
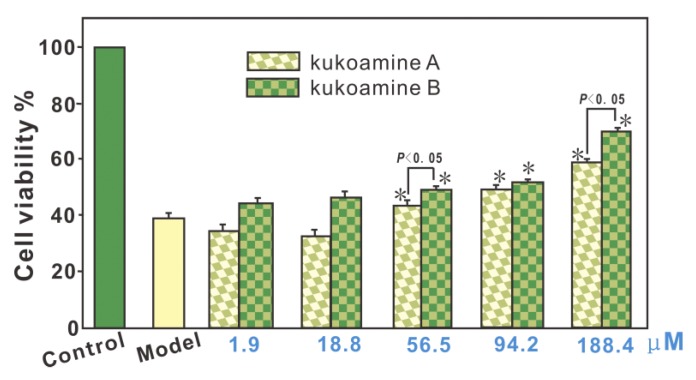
Protective effects of kukoamine A and B against Fenton-induced damage in bmMSCs, as measured in the MTT assay. These data represent the mean ± SD (*n* = 5). * *p* < 0.05 vs. model. The Fenton reagent (FeCl_2_ *plus* H_2_O_2_) was used to generate •OH radicals. MTT, 3-(-dimethylthiazol-2-yl)-2,5-diphenyltetrazolium bromide.

It has been reported that oxidative damage lowers the viability of bmMSCs and limits their transplantation in clinical applications [17]. Oxidative damage is well known to result from the accumulation of ROS such as H_2_O_2_ molecules and •OH radicals. H_2_O_2_ molecules can transform into •OH radicals via the Fenton reaction, under the catalysis of Fe^2+^ [18]. Thus, the mixture of H_2_O_2_ and Fe^2+^ is sometimes termed as the Fenton reagent. In fact, the Fenton reagent can also be found in bmMSCs [19]. In our study, the Fenton reagent was used to induce oxidative damage of bmMSCs. These Fenton reagent-damaged bmMSCs were assigned as the model group. As seen in Figure 2, the model group showed only 39.6 ± 0.6% cellular viability, and the control group (without Fenton reagent treatment) displayed 100% cellular viability. However, when the Fenton reagent-damaged bmMSCs were further incubated with kukoamine A (or B) at 56.5–188.4 μM, the cellular viabilities were concentration-dependently restored. These data suggest that both kukoamines possess cytoprotective effects in Fenton-damaged bmMSCs. Such cytoprotective effects can be partly responsible for the neuroprotective effects mentioned above, and predict that kukoamines may be a therapeutic candidate in bmMSCs transplantation for clinical applications in nervous system diseases.

**Figure 3:**
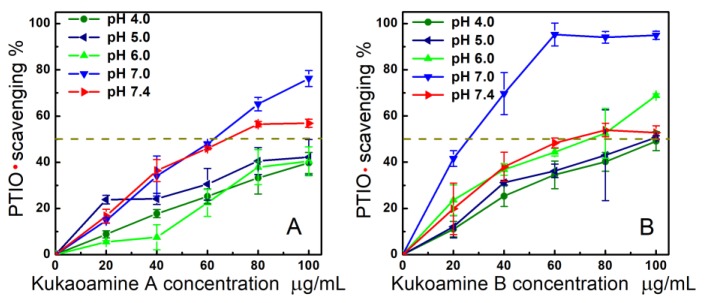
Concentration-response curves for kukoamine A (**A**) and kukoamine B (**B**) in PTIO• scavenging at various pH values (pH 4.0, 5.0, 6.0, 7.0, and 7.4) (Trolox concentration response was measured only at pH 7.4 as a positive control. The concentration-response curves of Trolox are shown in Supplementary 1. Each value is expressed as the mean ± SD, *n* = 3; The IC_50_ values were detailed in Table 1).

Our previous study pointed out that such cytoprotective effects are usually related to antioxidant (especially ROS-scavenging) effects [20]. In order to explore this possibility, we performed a PTIO• assay, a simple method newly developed by our team [21]. Similar to cellular ROS (e.g., •OH or •O_2_^−^), a PTIO• radical is an oxygen-centered radical. However, it can exist stably in aqueous solution (or buffer), and PTIO• scavenging can be easily measured in chemical solutions (or buffer). As shown in Figure 3, both kukoamine A and B showed concentration-dependent increases in PTIO• scavenging up to 100 µg/mL. These data indicated that kukoamine A and B could directly scavenge ROS, and direct ROS-scavenging may be one of the mechanisms in their antioxidant action.

As shown in Figure 3B and Table 1, under different pH values (pH 4.0, 5.0, 6.0, 7.0, and 7.4), each kukoamine presented different dose-response curves and different IC_50_ values. In general, lower buffer pH values resulted in higher observed IC_50_ values. Such pH effects suggested that the PTIO•-scavenging actions of kukoamines may be involved in the proton-transfer (H^+^-transfer) pathway, and kukoamines have proton-transfer ability during direct ROS-scavenging processes. This is partially supported by the fact that, in the reaction of PTIO• with ascorbic acid, proton-transfer (or •H-transfer) signal was observed by HPLC-MS [21]. Kukoamine A and B, however, are thought to partly ionize to give rise to H^+^ ion; and massive H^+^ ion in solution may suppress the H^+^ ionization from phenolic kukoamines to lower the antioxidant potential [22].

**Figure 4:**
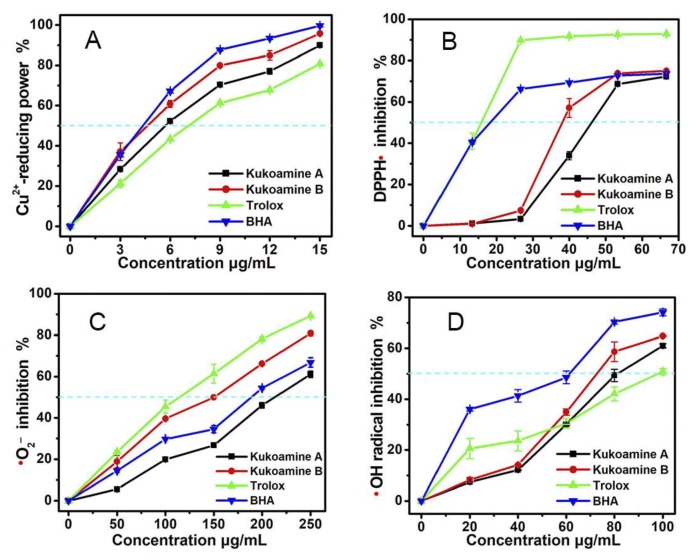
The antioxidant effects of kukoamine A and B in various assays: (**A**) Cu^2+^-reducing assay; (**B**) DPPH•-scavenging assay; (**C**) •O_2_^−^-scavenging assay; (**D**) •OH-scavenging assay. (Each value is expressed as the mean ± SD, *n* = 3; Trolox was the positive control.).

On the other hand, even in the pH 4.0 or pH 5.0 buffers, kukoamines still exhibited good concentration-dependent scavenging abilities (Figure 3). PTIO• scavenging at below pH 5.0 has been proposed to be an electron-transfer process as demonstrated by cyclic voltammetry [23]. Therefore, kukoamine A and B have electron-transfer potential. This possibility was further confirmed by evidence from the Cu^2+^-reducing assay (Figure 4A), an electron-transfer-based metal reducing reaction.

Of course, the electron and proton may be transferred together; a mechanism known as the hydrogen-atom-transfer (HAT) process [24]. To test this possibility, DPPH• scavenging was measured. In the DPPH• scavenging reaction, the HAT pathway has been proven to be indispensable, despite the fact that electron-transfer, sequential proton loss electron transfer (SPLET), proton-coupled electron transfer (PCET), electron-transfer, and radical adduct formation (RAF) may also take place [25,26,27,28,29]. The fact that kukoamine A and B could effectively scavenge DPPH• (Figure 4B), implies that kukoamines possessed HAT potential as a direct antioxidant mechanism. After interacting with the HAT pathway, kukoamines may further react with DPPH• causing a RAF reaction [30].

**Figure 5:**
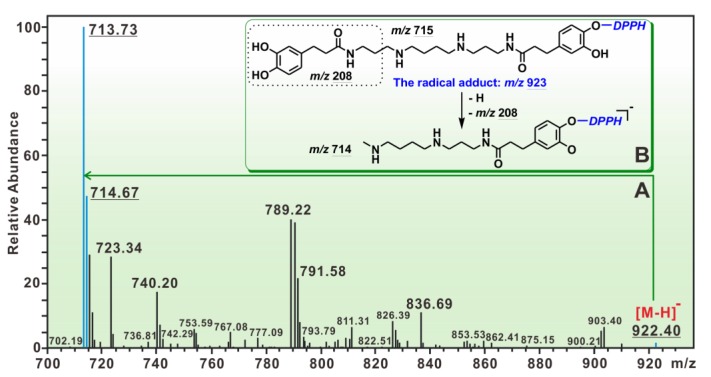
HPLC-MS results of the reaction mixture of kukoamine A with DPPH• radicals: (**A**) main RAF peaks of kukoamine A with DPPH•; (**B**) MS elucidation of radical adduct of kukoamine A with DPPH•. (The MS spectra of kukoamine A and relevant MS spectra elucidation are shown in Supplementary 2).

To explore the RAF possibility, the reaction mixture of kukoamine A with DPPH• was investigated using HPLC–ESI–MS/MS analysis. Two MS peaks (*m*/*z* 922, 713) relevant to RAF have been found (Figure 5A). The peak at *m*/*z* 922 was proposed to be the molecular ion peak of the kukoamine A-DPPH• adduct; while the peak at *m*/*z* 713 was its fragment (Figure 5B). However, the observed peak strengths were very low. In the product of kukoamine B with DPPH•, however, no RAF was observed by HPLC–ESI–MS/MS analysis. These data suggest that the RAF reaction served as a minor antioxidant mechanism [30].

**Figure 6:**
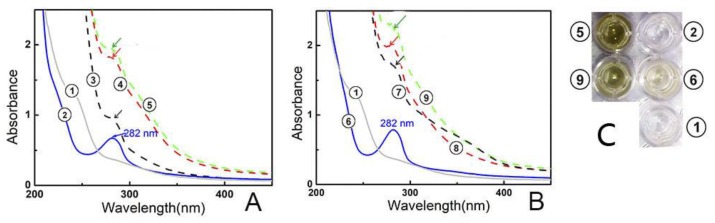
The experimental results of UV-vis-spectra analysis of Fe^2+^-chelation with kukoamine A and B. (**A**) kukoamine A; (**B**) kukoamine B; (**C**) solution appearances. (①151.0 mmol/L Fe^2+^; ② 0.2 mmol/L kukoamine A; ③ reaction mixture of 151.0 mmol/L Fe^2+^ with 0.2 mmol/L kukoamine A for 0 min; ④ reaction mixture of 151.0 mmol/L Fe^2+^ with 0.2 mmol/L kukoamine A for 30 min; ⑤ reaction mixture of 151.0 mmol/L Fe^2+^ with 0.2 mmol/L kukoamine A for 60 min; ⑥ 0.2 mmol/L kukoamine B; ⑦ reaction mixture of 151.0 mmol/L Fe^2+^ with 0.2 mmol/L kukoamine B for 0 min; ⑧ reaction mixture of 151.0 mmol/L Fe^2+^ with 0.2 mmol/L kukoamine B for 30 min; ⑨ reaction mixture of 151.0 mmol/L Fe^2+^ with 0.2 mmol/L kukoamine B for 60 min).

**Figure 7:**
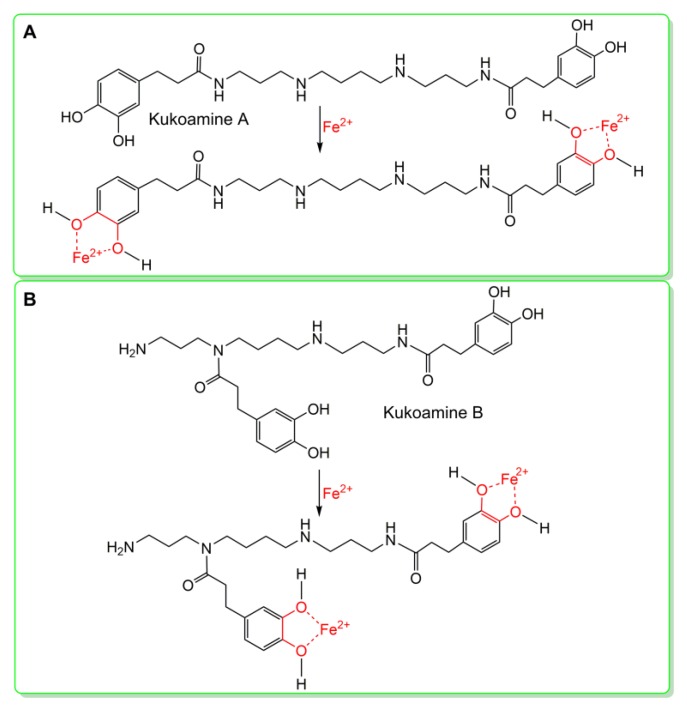
The proposed chelation reactions of kukoamine A (**A**) and kukoamine B (**B**) with excessive Fe^2+^.

Besides direct ROS-scavenging potential, indirect antioxidant potential was also studied. The so-called indirect antioxidant potential is actually Fe^2+^-chelation [24], because chelating catalyst Fe^2+^ can greatly reduce •OH generation. In fact, modulation of metal homeostasis and the inhibition of the Fenton reaction have been considered as one possible mechanism [31]. To test the Fe^2+^-chelation ability, the mixtures of kukoamines with excessive Fe^2+^ were analyzed using UV-vis spectra. As seen in Figure 6A, the reaction mixture of kukoamine A and Fe^2+^ generated stronger peak absorption than kukoamine A or Fe^2+^ solution alone. Meanwhile, the solution turned green in color. Similar changes were also observed in the experiment with kukoamine B (Figure 6B). These changes clearly indicated the occurrence of a Fe^2+^-chelating reaction. Thus, there is a possible Fe^2+^-chelation (i.e., indirect antioxidant) during the antioxidant process. Previous reports [31] show the dihydrocaffeoyl moiety acts as the ligand in Fe^2+^-chelation, thus, Fe^2+^-chelation reactions can be proposed as shown in Figure 7.

Based on the above mechanistic studies, it can be presumed that kukoamines may exert their ROS-scavenging actions via multiple mechanisms. These mechanisms include direct pathways (such as proton-transfer, electron-transfer, HAT, and RAF), and an indirect pathway (i.e., Fe^2+^-chelation). This presumption is further supported by the findings of •OH-scavenging and •O_2_^−^-scavenging assays, two multi-pathways-based radical reactions [32,33,34]. As seen in Figure 4C,D, each of the kukoamines could successfully scavenge •OH and •O_2_^−^ radicals, which are two free radicals occurring in Fenton-treated cells. 

However, in the above five spectrophotometry-based antioxidant assays (i.e., PITO•-scavenging assay, Cu^2+^-reducing assay, DPPH•-scavenging assay, •OH-scavenging assay, and •O_2_^−^-scavenging assay), there are substantial differences in antioxidant levels between kukoamine A and B. Generally, kukoamine B presented lower IC_50_ values than kukoamine A (Table 1), meaning that kukoamine B has higher antioxidant potentials than kukoamine A via proton-transfer, electron-transfer, and HAT mechanisms. 

As mentioned above, kukoamine A and B are positional isomers. The only difference between them is the dihydrocaffeoylation position. Thus, the difference in antioxidant levels can only be attributed to the positional isomerization. Such positional isomeric effect is assumed to be from the interaction between functional groups, such as a macrocycle by a hydrogen bridge between the hydrogen of the chelate and middle N-atom, or a field inductive effect between the dihydrocaffeoyl moiety and middle N-atom. Field inductive effects, however, have been reported to affect hydrogen abstraction (i.e., hydrogen-atom-transfer) or proton dissociation (i.e., proton-transfer) of phytophenols [35,36,37,38]. Of course, the detailed interactions may be complicated and require further investigation in the future. 

Moreover, such positional isomeric effects were found to affect Fe^2+^-chelation capacity, an indirect antioxidant mechanism. As seen in Figure 6, solution ⑦ exhibited a stronger UV-vis spectral peak than solution ③, while solution ⑤ displayed a stronger UV-vis spectral peak and darker color than solution ⑨. These differences indicated that kukoamine B had kinetic and thermodynamic advantages over kukoamine A in the Fe^2+^-chelation reaction. As shown in Figure 1, in the linear-chain kukoamine A molecule, the two dihydrocaffeoyl moieties are too distant to jointly chelate Fe^2+^, hence they can only chelate Fe^2+^ individually; In the branched-chain kukoamine B molecule, the two dihydrocaffeoyl moieties can not only individually but also jointly chelate Fe^2+^, because the two dihydrocaffeoyl moieties can possibly surround some Fe^2+^ in solution. As a result, kukoamine B exhibited higher Fe^2+^-chelating levels than did kukoamine A. 

The advantages in direct and indirect antioxidant potentials make kukoamine B superior to kukoamine A in terms of cytoprotective effects (Figure 2).

## 3. Materials and Methods

### 3.1. Chemicals

Kukoamine A (CAS 75288-96-9, C_28_H_42_N_4_O_6_, MW. 530.7, 97%, Supplementary 3) and kukoamine B (CAS 164991-67-7, C_28_H_42_N_4_O_6_, MW. 530.7, 97%, Supplementary 3) were obtained from Chengdu Biopurify Phytochemicals Ltd. (Chengdu, China). DPPH• (1,1-diphenyl-2-picrylhydrazyl radical), Trolox (±-6-hydroxyl-2,5,7,8-tetramethlyhromane-2-carboxylic acid), pyrogallol and neocuproine (2,9-dimethyl-1,10-phenanthroline) were purchased from Sigma Aldrich Trading Co. (Shanghai, China); d-2-deoxyribose and ABTS [2,2′-azino-bis(3-ethyl-benzothiazoline-6-sulfonic acid diammonium salt)] were obtained from Amresco Co. (Solon, OH, USA). Methanol and water were of HPLC grade. Dulbecco’s modified Eagle’s medium (DMEM), fetal bovine serum (FBS), and 3-(4,5-dimethylthiazol-2-yl)-2,5-diphenyl (MTT) were purchased from Gibco (Grand Island, NY, USA); CD44 and Proteinase K were purchased from Wuhan Boster Co., Ltd. (Wuhan, China). All other chemicals used were of analytical grade.

Four-week old Sprague-Dawley (SD) rats were obtained from the Animal Center of Guangzhou University of Chinese Medicine. These experiments were performed under the supervision of the Institutional Animal Ethics Committee of the Guangzhou University of Chinese Medicine (Approval number 20170306A). 

### 3.2. Protective Effect against Fenton-Induced Damage to bmMSCs (MTT Assay)

**Figure 8:**
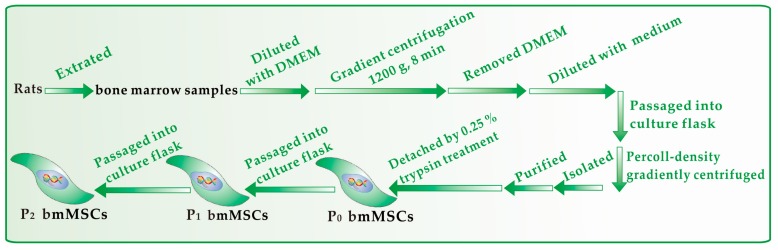
Experimental procedures for the preparation and culture of bmMSCs.

**Figure 9:**
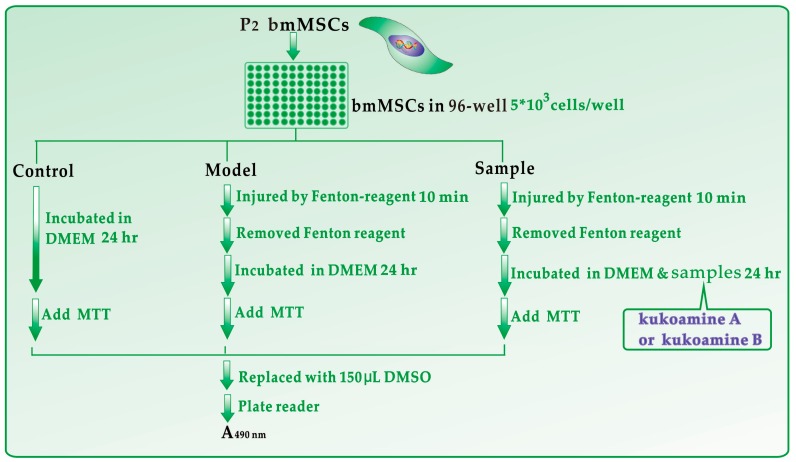
Experimental procedures for the MTT assay. Each test was repeated in five independent wells. Fenton reagent: FeCl_2_ (100 μM) followed by H_2_O_2_ (50 μM); MTT: 5 mg/mL in PBS, 20 μL; PE-1420 Bio-Kinetics reader: Bio-Kinetics Corporation, Sioux Center, IA, USA.

bmMSCs culture was carried out according to our previous report [39] with slight modifications. bmMSCs at passage 3 were detected for cell homogeneity based on CD44 expression by flow cytometry (Figure 8). The protective effect of kukoamines against •OH radical-induced bmMSCs damage was evaluated using the MTT assay [40]. The experimental protocol is briefly illustrated in Figure 9.

### 3.3. PTIO•-Scavenging Assay

**Figure 10:**
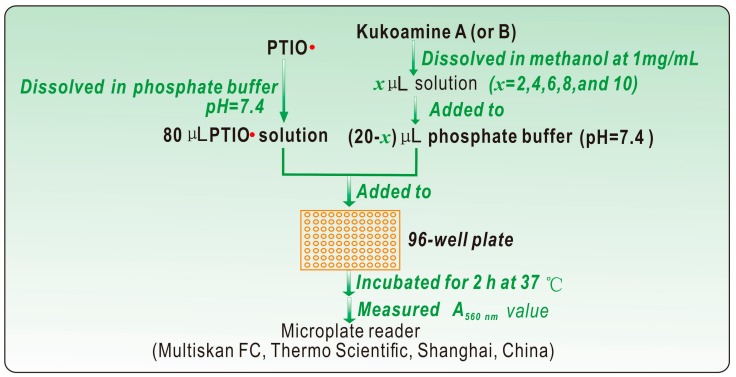
Experimental procedures for the PTIO•-scavenging assay. The above experiment was repeated using phosphate buffers with different pH values, including pH 4.0, 5.0, 6.0, 7.0 and 7.4.

The PTIO•-scavenging assay was conducted based on our previously published method [21]. The experimental procedures are briefly described in Figure 10. The PTIO• inhibition percentage was calculated as follows:

(1) $$Inhibition\%=\frac{A_{0}-A}{A_{0}}\times 100\%$$

where A_0_ is the absorbance at 560 nm of the control without the sample, and A is the absorbance at 560 nm of the reaction mixture with the sample. The above experiment was repeated using phosphate buffers with different pH values (including pH 4.0, 5.0, 6.0, and 7.0).

### 3.4. Cupric Ions (Cu^2+^) Reducing Antioxidant Power (CUPRAC) Assay

The CUPRAC assay was adapted from Apak’s method [41]. In brief, 125 μL CuSO_4_ aqueous solution (10 mM), 125 μL neocuproine ethanol solution (7.5 mM) and (750 − x) μL CH_3_COONH_4_ buffer solution (100 mM, pH 7.0) were added to test tubes followed by different volumes of samples (0.1 mg/mL, x= 30–150 μL). Then, the total volume was adjusted to 1000 μL with the buffer and mixed vigorously. Absorbance against a buffer blank was measured at 450 nm after 30 min. The relative reducing power of the sample as compared with the maximum absorbance was calculated using the formula:

(2) $$Relative\;reducing\;effect\%=\frac{A-A_{min}}{A_{max}-A_{min}}\times 100\%$$

where A_max_ is the maximum absorbance at 450 nm and A_min_ is the minimum absorbance in the test. A is the absorbance of the sample. 

### 3.5. DPPH•-Scavenging Assay

The DPPH•-scavenging activity was evaluated by the method [42]. Briefly, 100 μL of DPPH• solution (0.1 mM) was mixed with 50 μL sample ethanol solution of various concentrations. The mixture was kept at room temperature for 30 min, and then the absorbance was measured at 519 nm against ethanol (as a blank). The DPPH• inhibition percentage was calculated based on the formula presented in Section 3.3.

### 3.6. HPLC–ESI–MS/MS Analysis of the Reaction Products of Kukoamine A with DPPH•

Methanol solutions of kukoamine A and DPPH• radical were mixed with each other at a molar ratio of 1:2, and the resulting mixture was incubated for 30 min at room temperature. The product mixture was then filtered through a 0.22-μm filter and analyzed by a HPLC-ESI-MS/MS system equipped with a C_18_ column (TC-C_18_, 250 × 4.6 mm, 5 μm, Agilent Technologies Co., Beijing, China). The mobile phase was utilized for the separation and consisted of a mixture of methanol (phase A) and water (phase B). The column was eluted at a flow rate of 0.3 mL/min with the following elution program: 0–18 min, 75–84.5% A; 18–40 min, 85% A; 40–45 min, 75% A. The injection volume was 5 μL and the detection wavelength was set to 227 nm. Further analysis was performed on a triple quadrupole mass spectrometer (TSQ Quantum Access MAX, Thermo Fisher Scientific Inc., Waltham, MA, USA) equipped with an electrospray ionisation (ESI) source, which was run in negative mode. The scan range was 100–1000 *m*/*z*. ESI parameters were optimized with direct infusion of dansylated amine mixture by an external syringe and set as follows: capillary, +2.5 kV; nebulizer pressure, 30 psi; dry gas flow, 5 arb; dry gas temperature, 180 °C. Argon was applied as the collision gas, and the collision energy was set to 25–35 eV to provide some structural information and to focus ion flux. High purity nitrogen was used both as a nebulizer gas and a drying gas. Reactants kukoamine A and DPPH• radical were also comparatively measured under the same conditions.

### 3.7. Fe^2+^-Chelating Assay by Ultraviolet-Visible (UV-Vis) Spectra Analysis

The Fe^2+^-chelating ability was assessed by UV-Vis spectroscopy [28]. In brief, 100 μL sample methanol solution (1 mg/mL) and 300 μL FeCl_2_•4H_2_O aqueous solution (100 mg/mL) were added to 600 μL of methanol-water (1:1, *v*/*v*), and mixed well. The resulting mixture was subsequently scanned using a UV-vis spectrophotometer (Unico 2600A, Shanghai, China) from 200–800 nm in an hour. Next, 200 μL of the supernatant was transferred to a 96-well plate and photographed using a camera.

### 3.8. Deoxyribose Degradation Assay for •OH-Scavenging

The measurement of •OH radical-scavenging was conducted according to our previously published method [43]. In brief, the sample ethanol solution (4 mg/mL, 9–45 μL) was separately added into tubes. After evaporating the sample solutions in the tubes to dryness, 400 μL of phosphate buffer (0.2 M, pH 7.4) was added to the sample residue. Then, 50 μL deoxyribose (50 mM), 50 μL Na_2_EDTA (1 mM), 50 μL FeCl_3_ (3.2 mM), and 50 μL H_2_O_2_ (50 mM) were added. The reaction was initiated by mixing 50 μL ascorbic acid (1.8 mM) and the total volume of the reaction mixture was adjusted to 800 μL with buffer. After incubation at 50 °C for 20 min, the reaction was terminated by addition of 250 μL trichloroacetic acid (10%, *w*/*w*). The color was then developed by addition of 150 μL 2-thiobarbituric acid (5%, in 1.25% NaOH aqueous solution) and heated in an oven at 105 °C for 15 min. The mixture was cooled and absorbance was measured at 530 nm (Unico 2100 spectrophotometer, Shanghai, China) against the buffer (as a blank). The hydroxyl radical scavenging activity was calculated based on the formula presented in Section 3.3.

### 3.9. Superoxide Anion Radical (•O_2_^−^)-Scavenging Assay

The superoxide anion radical (•O_2_^−^)-scavenging assay method was developed by our laboratory [44]. Briefly, the sample was dissolved in ethanol at 1 mg/mL. The sample solution (x μL, where x = 0, 50, 100, 150, 200 and 250 μL) was mixed with 2950-x μL Tris-HCl buffer (0.05 M, pH 7.4) containing Na_2_EDTA (1 mM). When 50 μL pyrogallol (60 mM in 1 mM HCl) was added, the mixture was shaken at room temperature immediately. The absorbance of the mixture at 325 nm was measured (Unico 2100, Shanghai, China) against Tris-HCl buffer as a blank every 30 s for 5 min. The •O_2_^−^ scavenging ability was calculated as:

(3) $$\mathrm{Inhibition}\%=\frac{\left(\frac{A_{325\mathrm{nm},\;\mathrm{control}}}{T}\right)-\left(\frac{A_{325\mathrm{nm},\;\mathrm{sample}}}{T}\right)}{\left(\frac{A_{325\mathrm{nm},\;\mathrm{control}}}{T}\right)}\times 100\%$$

Here, ΔA_325nm, control_ is the increase in A_325nm_ of the mixture without the sample and ΔA_325nm, sample_ is that with the sample; T = 5 min. 

### 3.10. Statistical Analysis

Each experiment was performed in triplicate; the data were recorded as mean ± SD (standard deviation). The dose-response curves were plotted using Origin 6.0 professional software (OriginLab, Northampton, MA, USA). The IC_50_ value was defined as the final concentration of 50% radical inhibition (or relative reducing power) [45]. Statistical comparisons were made by one-way ANOVA to detect significant differences using SPSS 13.0 software (SPSS Inc., Chicago, IL, USA) for windows. *p* < 0.05 was considered to be statistically significant.

## 4. Conclusions

Two isomeric phenolic polyamines, kukoamine A and B, can protect bmMSCs from Fenton-induced damage through direct antioxidant pathways (including electron-transfer, proton-transfer, hydrogen-atom-transfer, and RAF), and an indirect antioxidant pathway (i.e., Fe^2+^-chelation). In these pathways, kukoamine B always exhibits higher antioxidant levels than kukoamine A. Thus, kukoamine B is superior to kukoamine A in cytoprotection. These differences can ultimately be attributed to positional isomeric effects between the two kukoamines.

## Figures and Tables

**Table 1:** The IC50 values of kukoamine A and B in various antioxidant assays.

Assays	kukoamine A μg/mL (μM)	kukoamine B μg/mL (μM)	Trolox
PTIO•-scavenging (pH 7.4)	74.9 ± 2.3 (140.1 ± 4.3 ^a,B^)	78.1 ± 2.6 (148.6 ± 5.0 ^a,B^)	83.8 ± 5.4 (333.3 ± 21.5 ^b^)
PTIO•-scavenging (pH 7.0)	63.1 ± 1.4 (118.9 ± 2.6 ^b,A^)	15.6 ± 3.2 (29.4 ± 6.1 ^a,A^)	ND
PTIO•-scavenging (pH 6.0)	163.7 ± 14.5 (308.4 ± 27.2 ^b,D^)	69.0 ± 4.1 (130.0 ± 7.6 ^a,B^)	ND
PTIO•-scavenging (pH 5.0)	147.3 ± 27.0 (277.6 ± 51.0 ^b,C^)	94.4 ± 13.4 (177.9 ± 25.3 ^a,C^)	ND
PTIO•-scavenging (pH 4.0)	162.7 ± 3.3 (306.5 ± 6.1 ^b,D^)	110.8 ± 9.4 (208.8 ± 17.7 ^a,D^)	ND
Cu^2+^-reducing	5.5 ± 0.2 (10.3 ± 0.3 ^b^)	4.7 ± 0.2 (8.9 ± 0.3 ^a^)	7.04 ± 0.1 (28.1 ± 0.5 ^d^)
DPPH•-scavenging	46.0 ± 0.4 (86.6 ± 0.7 ^c^)	39.1 ± 0.4 (73.7 ± 0.7 ^b^)	14.5 ± 0.8 (58.1 ± 3.0 ^a^)
•O_2_^-^-scavenging	213.0± 2.7 (401.4 ± 5.0 ^b^)	147.6 ± 1.8 (278.2 ± 3.4 ^a^)	111.9 ± 0.6 (447.2 ± 2.3 ^c^)
•OH-scavenging	89.0 ± 1.7 (167.7 ± 3.2 ^b^)	78.6 ± 4.7 (146.9 ± 8.8 ^a^)	101.6 ± 4.0 (405.8 ± 16.0 ^c^)

The IC_50_ value (in μg/mL unit) was defined as the final concentration of 50% radical inhibition or relative reducing power, calculated by linear regression analysis, and expressed as the mean ± SD (*n* = 3). The linear regression was analyzed by Origin 6.0 professional software. The IC_50_ value in μM units, with different superscripts (^a^, ^b^, ^c^, or ^d^) in the same row, and (^A^, ^B^, ^C^, or ^D^) in the same column, are significantly different (*p* < 0.05). Trolox is the positive control. N.D., not detected.

## Supplementary Materials

The following are available online. Supplementary 1: Dose response curves of Trolox in PTIO assay; Supplementary 2: MS spectra of kukoamine A; Supplementary 3: Certificate analysis of kukoamine A and B.

## Abbreviations

The following abbreviations are used in this manuscript:

ABTS	2,2′-azino-bis(3-ethylbenzo-thiazoline-6-sulfonic acid diammonium salt)
bmMSCs	bone marrow-derived mesenchymal stem cells
DMEM	Dulbecco’s modified Eagle’s medium
DPPH•	1,1-diphenyl-2-picryl-hydrazl radical
FBS	fetal bovine serum
FRAP	ferric-reducing antioxidant power
HAT	hydrogen atom transfer
PTIO•	2-phenyl-4,4,5,5-tetramethylimidazoline-1-oxyl 3-oxide radical
ROS	reactive oxygen species
RAF	radical adduct formation
SD	standard deviation
TPTZ	2,4,6-tris(2-pyridyl-s-triazine)
Trolox	(±)-6-hydroxyl-2,5,7,8-tetramethlychromane-2-carboxylic acid
