# Supplementary material for: Antioxidant and Cytoprotective Effects of Kukoamines A and B: Comparison and Positional Isomeric Effect
Source: Molecules. 2018 Apr 21;23(4):973. doi: 10.3390/molecules23040973 (PMC6017596; doi:10.3390/molecules23040973)
Supplement: Supplementary file 1 [file molecules-23-00973-s001.zip › Suppls/Suppl. 2 MS spectra.pdf]

Supplemental Materials 2

Antioxidant and Cytoprotective Effects of Kukoamines A and B: Comparison and Positional Isomeric Effect

Xican Li [1, 2, *,†], Jian Lin [3, 4,†], Ban Chen [1, 2], Fei Li [5], Hong Xie [1, 2], Dongfeng Chen [3, 4, *]

[1] School of Chinese Herbal Medicine, [2] Innovative Research & Development Laboratory of TCM, [3] School of Basic Medical Science, [4]The Research Center of Basic Integrative Medicine, Guangzhou University of Chinese Medicine, [5] Jinan Municipal Hospital of Traditional Chinese Medicine, Jinan 250012, China.

[*] Corresponding author. E-mail: lixican@126. com(X.L.); chen888@gzucm.edu.cn (D.C.)

[†] Equal contributors

Postal addresses: Guangzhou University of Chinese Medicine, No.232, Waihuan East Road, Guangzhou Higher Education Mega Center, Panyu District, Guangzhou, 510006, China

Homepage: http://www.researchgate.net/profile/Xican_Li

Tel: +86 20 39358076

Fax: +86 20 38892697

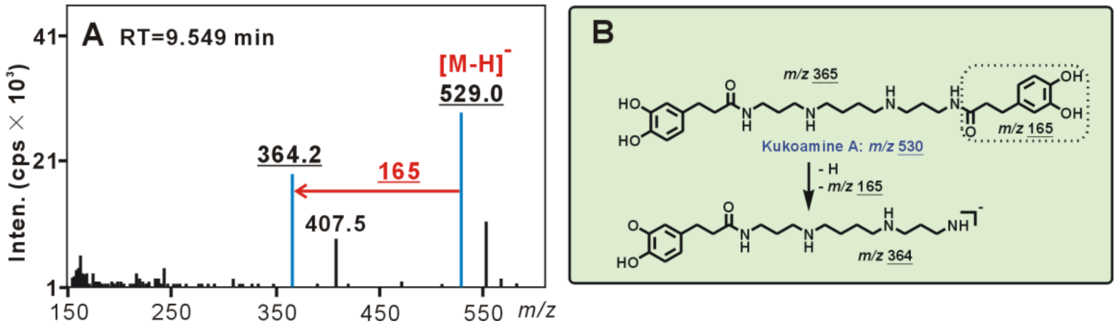

Suppl. 3 The MS spectra Kukoamine A determined by our instrument (HPLC-ESI-MS/MS system, TSQ Quantum Access MAX, Thermo Fisher Corp. USA) (A) and elucidation of Kukoamine A (B)
