# Supplementary material for: Antioxidant and Cytoprotective Effects of Kukoamines A and B: Comparison and Positional Isomeric Effect
Source: Molecules. 2018 Apr 21;23(4):973. doi: 10.3390/molecules23040973 (PMC6017596; doi:10.3390/molecules23040973)
Supplement: Supplementary file 1 [file molecules-23-00973-s001.zip › Suppls/Suppl. 3 Analysis certificates.pdf]

Supplemental Materials 3- Certificates of analysis of Kukoamine A & B

# Antioxidant and Cytoprotective Effects of Kukoamines A and B: Comparison and Positional Isomeric Effect

**Xican Li [1, 2, *,†], Jian Lin [3, 4,†], Ban Chen [1, 2], Fei Li [5], Hong Xie [1, 2], Dongfeng Chen [3, 4, *]**

[1] School of Chinese Herbal Medicine, [2] Innovative Research & Development Laboratory of TCM, [3] School of Basic Medical Science, [4]The Research Center of Basic Integrative Medicine, Guangzhou University of Chinese Medicine, [5] Jinan Municipal Hospital of Traditional Chinese Medicine, Jinan 250012, China.

[*] Corresponding author. E-mail: lixican@126. com(X.L.); chen888@gzucm.edu.cn (D.C.)

[†] Equal contributors

**Postal addresses:** Guangzhou University of Chinese Medicine, No.232, Waihuan East Road, Guangzhou Higher Education Mega Center, Panyu District, Guangzhou, 510006, China

**Homepage:** http://www.researchgate.net/profile/Xican_Li

**Tel:** +86 20 39358076

**Fax:** +86 20 38892697

# Kukoamine A 0.1mg/mL

实验时间：2017-12-27,19:28:10
谱图文件:C:\浙大智达\N2000\样品\Kukoamine A.mdy

实验者：
报告时间：2017-12-27,19:28:23
积分方法:面积归一法

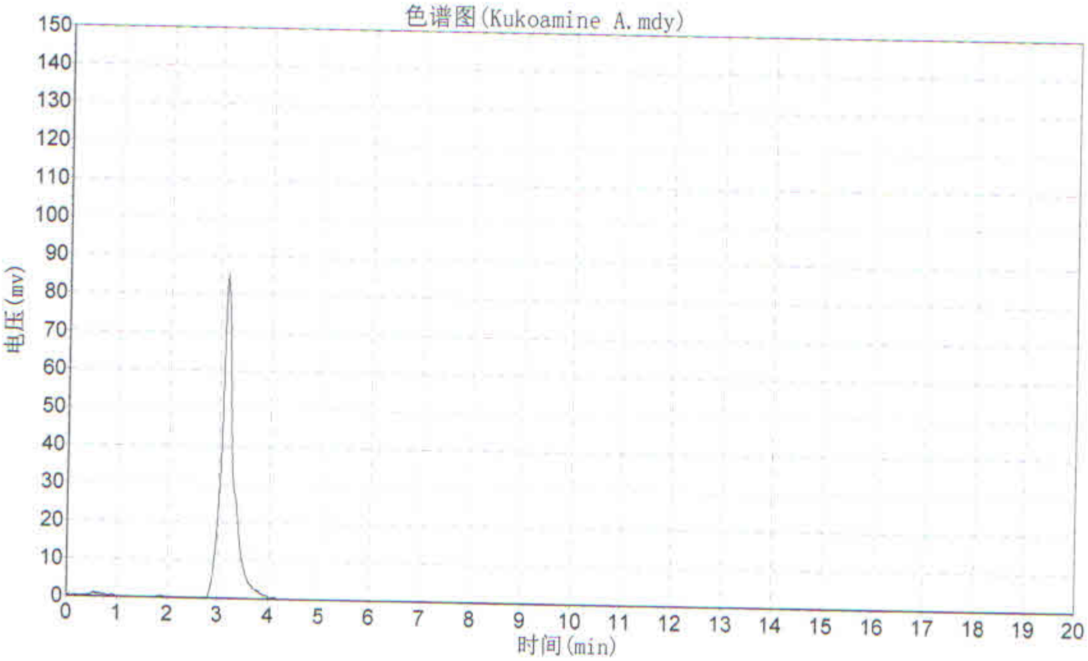


## 分析结果表

| 峰号 | 峰名 | 保留时间 | 峰高 | 峰面积 | 含量 |
|---|---|---|---|---|---|
| 1 | | 3.140 | 84752.164 | 1447065.125 | 97.7033 |
| 2 | | 7.207 | 2707.512 | 34015.887 | 2.2967 |
| 总计 | | | 87459.677 | 1481081.012 | 100.0000 |

Column Type: Agilent 5 TC-C18 250*4.6 mm column
Mobile Phases: Acetonitrile 10 %, 0.1% phosphoric acid in water 90%
Flow Rate: 1 mL/min
Detection Mode and Setting: UV280nm
Sample Diluent(s): Water
Sample Concentration: Approx 100μg/ml
Injection Volume: 10μl
Calculation: Area percent normalization

# 产品分析证书
## Certificate of Analysis

中文名称：地骨皮乙素

**English Name**：Kukoamine B
**别名(Alias):**

**产品编码(Cat. No.):**BP0834
**CAS Number: 164991-67-7**
**分子式(M. F.):** C28H42N4O6
**分子量(M. W.)**: 530.666

**批号(Batch No.):** PRF7090543
**报告日期(Report date): 2016/9/5**

**Structure**

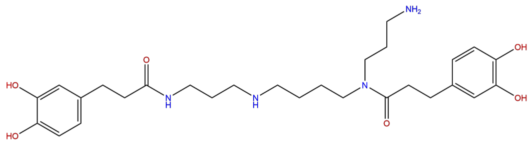

**检验结果（Analytical result）：**

| 检验项目（Test Item） | 检验指标（Specifications） | 检验结果（Results） |
|---|---|---|
| 外观Appearance | Brown powder | Brown powder |
| 干燥失重Loss on drying | ＜3.0% | 1.6% |
| 纯度Purity (HPLC-DAD,280nm)* | ≥98.0% | 98.37% |
| 质谱Mass | 530.666±1 | Conforms |
| 核磁NMR | Comply with the structure | Conforms |

*** 色谱图见附件（Please find HPLC chromatography attached.）**

贮存条件（Storage）:2~8℃
复测期（Retest date）:two years (2018-09-04) under conditions list above.
备注（Remarks）：如遇质量问题，请于收到产品之日起 15 日内与我们联系。
In case of quality issue, please contact us within 15 days after receipt of the product.

QC: Zhang Ling
Date: 2016年9月5日

QA: Wu Qi
Date: 2016年9月5日

Tel：+86-28-82633987　Fax：+86-28-82633165
http://www.biopurify.com　Email：sales@biopurify.com　biopurify@gmail.com

## SAMPLE INFORMATION

| | | | |
|---|---|---|---|
| Sample Name: | Kukoamine B | Acquired By: | System |
| Sample Type: | Unknown | Sample Set Name: | |
| Vial: | 80 | Acq. Method Set: | Kukoamine B |
| Injection #: | 1 | Processing Method: | sample |
| Injection Volume: | 25.00 ul | Channel Name: | 280.0nm |
| Run Time: | 25.0 Minutes | Proc. Chnl. Descr.: | PDA 280.0 nm |
| Date Acquired: | 2016-9-5 15:30:07 CST | | |
| Date Processed: | 2016-9-5 16:00:00 CST | | |

### Auto-Scaled Chromatogram

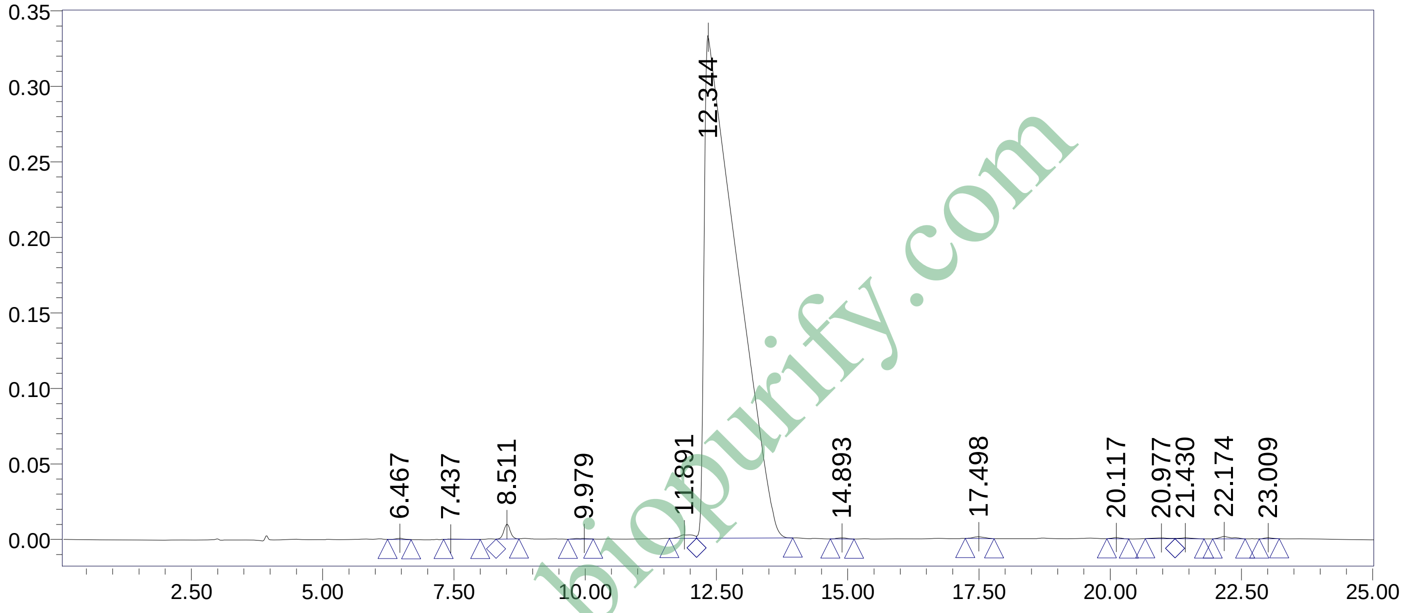


www.biopurify.com

### Peak Results

| | RT | Area | % Area | USP Plate Count | USP Resolution |
|---|---|---|---|---|---|
| 1 | 6.467 | 7698 | 0.05 | 9880.10 | |
| 2 | 7.437 | 6533 | 0.05 | 12612.73 | 3.49 |
| 3 | 8.511 | 84363 | 0.58 | 22082.73 | 4.16 |
| 4 | 9.979 | 7103 | 0.05 | 10648.22 | 4.82 |
| 5 | 11.891 | 43453 | 0.30 | | 5.07 |
| 6 | 12.344 | 14265565 | 98.37 | 1322.88 | 0.52 |
| 7 | 14.893 | 9442 | 0.07 | 25819.36 | 2.91 |
| 8 | 17.498 | 16548 | 0.11 | 35136.74 | 6.79 |
| 9 | 20.117 | 7544 | 0.05 | 89823.87 | 7.86 |
| 10 | 20.977 | 12222 | 0.08 | 33258.57 | 2.17 |

## Peak Results

| | RT | Area | % Area | USP Plate Count | USP Resolution |
|---|---|---|---|---|---|
| 11 | 21.430 | 9802 | 0.07 | 41853.68 | 0.94 |
| 12 | 22.174 | 24307 | 0.17 | 63089.30 | 1.77 |
| 13 | 23.009 | 7182 | 0.05 | 111995.08 | 2.45 |

www.biopurify.com
